# Ultra-sensitive detection of *Mycobacterium leprae*: DNA extraction and PCR assays

**DOI:** 10.1371/journal.pntd.0008325

**Published:** 2020-05-26

**Authors:** Fernanda Saloum de Neves Manta, Thyago Leal-Calvo, Suelen Justo M. Moreira, Brunna L. C. Marques, Marcelo Ribeiro-Alves, Patrícia S. Rosa, José Augusto C. Nery, Rita de Cássia Pontello Rampazzo, Alexandre Dias Tavares Costa, Marco Aurelio Krieger, Milton Ozório Moraes

**Affiliations:** 1 Laboratório de Hanseníase, Instituto Oswaldo Cruz—Fiocruz, Rio de Janeiro, RJ, Brazil; 2 Laboratório de Pesquisa Clínica em DST-AIDS, Instituto Nacional de Infectologia Evandro Chagas—Fiocruz, Rio de Janeiro, RJ, Brazil; 3 Instituto Lauro de Souza Lima, Bauru, SP, Brazil; 4 Instituto de Biologia Molecular do Paraná—Fiocruz, Curitiba, PR, Brazil; 5 Instituto Carlos Chagas, Fundação Oswaldo Cruz—Fiocruz, Curitiba, PR, Brazil; Lowell General Hospital, UNITED STATES

## Abstract

Leprosy urgently needs a precise and early diagnostic tool. The sensitivity of the direct (bacilli staining, *Mycobacterium leprae* DNA) and indirect (antibody levels, T cell assays) diagnostics methods vary based on the clinical form. Recently, PCR-based *M*. *leprae* DNA detection has been shown to differentially diagnose leprosy from other dermatological conditions. However, accuracy can still be improved, especially for use with less invasive clinical samples. We tested different commercial DNA extraction kits: DNeasy Blood & Tissue, QIAamp DNA Microbiome, Maxwell 16 DNA Purification, PowerSoil DNA Isolation; as well as in-house phenol-chloroform and Trizol/FastPrep methods. Extraction was performed on *M*. *leprae*-infected mouse footpads and different clinical samples of leprosy patients (skin biopsies and scrapings, lesion, oral and nasal swabs, body hair, blood on FTA cards, peripheral whole blood). We observed that the Microbiome kit was able to enrich for mycobacterial DNA, most likely due the enzymatic digestion cocktail along with mechanical disruption involved in this method. Consequently, we had a significant increase in sensitivity in skin biopsies from paucibacillary leprosy patients using a duplex qPCR targeting 16S rRNA (*M*. *leprae*) and 18S rRNA (mammal) in the StepOnePlus system. Our data showed that the presence of *M*. *leprae* DNA was best detected in skin biopsies and skin scrapings, independent of the extraction method or the clinical form. For multibacillary patients, detection of *M*. *leprae* DNA in nasal swabs indicates the possibility of having a much less invasive sample that can be used for the purposes of DNA sequencing for relapse analysis and drug resistance monitoring. Overall, DNA extracted with the Microbiome kit presented the best bacilli detection rate for paucibacillary cases, indicating that investments in extraction methods with mechanical and DNA digestion should be made.

## Author summary

Leprosy is difficult to diagnose since it is caused by a bacterium that does not grow *in vitro*. Bacilli direct detection or the presence of specific antibodies can vary greatly depending on the clinical form. *M*. *leprae* direct DNA detection can aid clinical diagnosis, although invasive skin biopsies are still necessary to detect the pathogen or histological features consistent with leprosy. Here we show that a kit combining mechanical and chemical lysis efficiently removes host DNA and enriches for *M*. *leprae* DNA, allowing better detection of paucibacillary cases. We believe our findings can contribute to improving disease diagnosis, as well as early detection and that could help monitoring strategies.

## Introduction

According to the World Health Organization, leprosy is a continuing public health problem [1]. Leprosy is an infectious disease of slow evolution that can manifest in different clinical forms with dermatological and neurological signs and symptoms. It is believed that the most likely mode of transmission and infection occurs by means of secretions from the upper respiratory tract [2–4]. In addition, wild red squirrels (*Sciurus vulgaris*) and armadillos (*Dasypus novemcinctus*) were found to harbor viable *M*. *leprae*, where the latter has been implicated in zoonotic transmission to humans [5,6]. More recently, there is one study showing that ticks (*Amblyomma sculptum*) may also have the potential to act as a reservoir and/or vector of leprosy [7].

Furthermore, frequent and prolonged interaction between infected individuals and susceptible subjects is a key factor for the transmission of the *Mycobacterium leprae* [1]. Although multidrug therapy is effective in treating the disease, it has proved ineffective in the control of transmission. Difficulties in early diagnosis thus contribute to continuous bacteria dissemination from undiagnosed patients. Intervention strategies to block transmission and appropriate care of infected individuals depends on early and reliable pathogen detection as soon as symptoms emerge [8]. As in many infectious diseases, rapid and precise diagnosis is key in the organization of public policies for disease control, which encompasses not only chemo- and immune- prophylaxis but also contact surveillance [9].

Molecular biology techniques have been widely applied in the diagnosis of infectious and parasitic diseases. In particular, PCR is often employed for probing the presence of pathogen DNA in patient samples [10].

Since 2006, our group has been developing studies in the field of molecular diagnosis, where real-time quantitative PCR (qPCR) has proved to be a promising tool for enhanced detection in difficult-to-diagnose cases, such as pure neural leprosy or indeterminate paucibacillary leprosy with leprosy-like skin lesions [11,12]. Thus, patients who have suspicious lesions or signs of leprosy without clear clinical bacteriological or histopathological confirmation are able to be diagnosed [13,14].

However, specificity and sensitivity still need to be improved, as it is known that the type of clinical sample can affect accurate *M*. *leprae* detection. In this regard, the qPCR technique has been applied using nucleic acids derived from the different clinical samples typically collected, such as skin smears, nerve biopsies, urine, oral or nasal swabs, blood, and skin lesions [15–20].

A critical pre-analytical step in nucleic acid detection assays is the extraction step. Determining the best method of extraction and processing prior to qPCR could enhance the detection of *M*. *leprae* in these more difficult samples. Many DNA isolation kits are commercially available [21–23], but each method varies in the yield and purity of the nucleic acid obtained. An ideal isolation procedure should optimize yield and purity whilst minimizing DNA degradation, and also be efficient in terms of cost, time, labor and supplies. Furthermore, it should be suitable for extracting multiple samples and generate minimal hazardous waste.

Here, we systematically evaluated the impact of the biological sample and the DNA extraction method on the efficacy of *M*. *leprae* detection in suspected leprosy patients. Our aim was to determine the best combination of these factors in improving qPCR results as a molecular diagnostic tool for leprosy. Undoubtedly, our data suggest that an extraction method that involves a combination of enzymatic digestion of host DNA and cell wall lysis with enzyme cocktail and mechanical rupture improves *M*. *leprae* DNA detection in qPCR.

## Methods

### Study design

**Fig 1:**
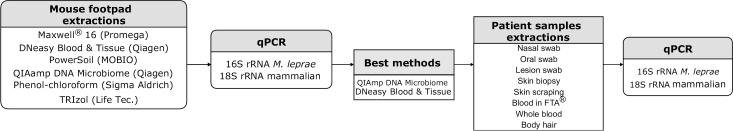
Overall workflow of the study.

The study was conducted between 2017 and 2018 in the Leprosy laboratory of the Oswaldo Cruz Foundation, Rio de Janeiro, Brazil and the workflow is summarized in Fig 1.

### Ethics statement

This study was approved by the Fiocruz ethics committee (CAAE: 38053314.2.0000.5248, number: 976.330–10/03/2015). An informed consent form was signed by all patients that were enrolled in the study. Animal work was granted approval (license number 219/11) by the Animal Welfare Committee of Sagrado Coração University (CEUA-USC) (São Paulo, Brazil). The University conducts animal care inspections and authorizations from Instituto Lauro de Souza Lima. All procedures were performed in accordance with the Brazilian guidelines for production, maintenance and use of animals in training or scientific research activities from CONCEA (from Portuguese, National Council for the Control of Animal Experimentation).

### Classification of leprosy patients

Different biological samples from the same leprosy patients were obtained at the outpatient clinic of the Oswaldo Cruz Institute, Fiocruz, Rio de Janeiro, Brazil. Patients were classified according to the Ridley-Jopling scale (R&J) and after sample collection, they were treated as multibacillary (MB) and paucibacillary (PB) as defined by WHO [24]. A total of 23 MB patients: 4 borderline–borderline (BB), 4 borderline lepromatous (BL) and 15 lepromatous, (LL); and 24 PB patients: 1 tuberculoid (TT) and 23 borderline tuberculoid (BT) were included in this study.

Sociodemographic and laboratory variables including age, gender and bacteriological index (BI) were collected for all patients (S1 Table).

### Sample collection

#### *Mycobacterium leprae* samples

*M*. *leprae*-infected footpads of athymic *nude* mice were obtained from the colony kept at the Instituto Lauro de Souza Lima, São Paulo, Brazil. Briefly, samples were received either in 70% ethanol or RNA Later (Ambion/Thermo Fisher Scientific) at room temperature or at 4°C. Total tissue from an individual mouse footpad was first carefully weighed; then cut into fragments of the same weight, which were randomly allocated to each extraction procedure. This way, every batch (different mice) yielded a given fragment of known mass in milligrams to be used in each extraction procedure in parallel.

#### Clinical samples

Eight clinical sample types were collected from the patients: skins scrapings (SS), skin biopsies (SB), whole blood (WB), blood on FTA paper (FTA), oral swabs (OS), nasal swabs (NS), skin lesion swabs (LS), and body hair (HR).

Skin scrapings were taken by making a small incision with a razor blade on the right earlobe followed by scraping of the region under tweezer pressure. Skin biopsies were collected using a 6-mm punch. Both the skin scrapings and skin biopsies were stored in 70% ethanol at -20°C until processing.

Whole blood samples were collected in blood collection tubes with sodium citrate (Vacuplast), while blood drops were dispensed onto FTA paper and stored at -20°C and 4°C, respectively, until processing.

For skin lesion swab samples, an eSwab (Copan Diagnostics) was gently brushed over the entire lesional area for 10 seconds. For the collection of a good amount of oral mucosa cells in oral swabs, the cheeks were gently exfoliated with an eSwab. For the nasal swab collection, an eSwab was inserted less than one centimeter into the nostril, rotated several times against the nasal wall, then repeated in other nostril using the same swab. After collection, the swabs tips were placed into collector tubes containing one mililiter of Amies Liquid Medium (ALM) (Copan Diagnostics) and one mililiter of 2X stabilization buffer solution (SBS—100 mM NaCl, 10 mM Tris HCl pH 7.5, 10 mM EDTA, 0.5% SDS, 0.1 mg/mL Proteinase K). Therefore, all swabs samples were stored in a final solution (ALM + 2X SBS) at 4°C.

Lastly, body hair around the skin lesions were gently collected with the aid of tweezers and stored in 70% ethanol at -20°C until processing.

Each of the collected samples was divided into equal parts to be used in the different DNA extraction kits.

#### DNA extraction from mouse footpads and clinical samples

DNA isolation from infected mouse footpads were tested with six DNA extraction methods: DNeasy Blood & Tissue Kit (QIAGEN), QIAamp DNA Microbiome Kit (QIAGEN), Maxwell 16 DNA Purification Kit (Promega), PowerSoil DNA Isolation Kit (QIAGEN/Mo Bio), *in-house* standard phenol-chloroform (Sigma Aldrich) plus FastPrep Lysing Matrix B (MP Biomedicals) and TRIzol (Thermo Fisher Scientific). After assessing the kits on the experimental mouse model, leprosy patient samples were tested with two chosen kits, DNeasy Blood & Tissue and QIAamp DNA Microbiome. All isolation procedures were performed according to the manufacturer’s standard protocols with minor modifications (S1 Text) and DNA was quantified on a NanoDrop D1000.

### Quantitative Polymerase Chain Reaction (qPCR)

The levels of *M*. *leprae* from mouse footpads and clinical samples were estimated through the amplification of the *M*. *leprae*-specific 16S rRNA target [12] in the TaqMan assay (Thermo Fisher Scientific). Another primer pair, which amplified a fragment of the mammalian 18S rRNA region, was used as an internal reference host DNA control (forward, 5´GAA ACT GCG AAT GGC TCA TTA AAT CA3´; reverse, 5´CCC GTC GGC ATG TAT TAG CTC T-3´; TaqMan probe, 5´TGG TTC CTT TGG TCG CTC GCT CC-3´). The two targets were analyzed in the same reaction, with samples submitted to a duplex reaction that simultaneously detects the bacteria and host DNA. All reactions were performed in triplicate on the same real-time PCR system (StepOnePlus, Applied Biosystems). Titrated *M*. *leprae* DNA was used as a positive control while molecular biology grade water was used in negative controls. For each reaction, 5 μL of a 10 ng/μL DNA stock solution (final 50 ng/reaction) were added for a 25 μL final volume containing TaqMan 2X master mix, 500 nM of each primer and 100 nM of each probe for 16S rRNA PCR assays and 160 nM of each primer and 40 nM of the probe for 18S rRNA PCR assay. A cycle threshold (Ct) cutoff value used was 0.05 for both targets. The sample was considered positive for *M*. *leprae* DNA when it exhibited a 16S rRNA Ct value ≤ 38.5 in at least two reactions of the triplicate and positive for host DNA if the 18S rRNA Ct value was between 13 and 33.

The standard curve (S1 Fig) was generated for each qPCR using eight dilutions (1 ng, 100 fg, 10 fg, 1 fg, 100 pg, 50 pg, 25 pg and 5 pg) of purified *M*. *leprae* DNA, and analyzed as described in Martinez et al. (2011) [12]. The number of genomes was calculated by interpolating sample Ct values with those from a dilution curve made from a known number of *M*. *leprae* genomes, where one *M*. *leprae* genome was assumed to weigh 3 fg [14]. After obtaining genome numbers from the standard curve, the amount of *M*. *leprae* was calculated as follows: [*M*. *leprae* DNA equivalents per reaction/amount of tissue DNA per reaction] ×10^3^, expressed as the number of *M*. *leprae* genomes per μg of tissue DNA [25,26].

### Statistical analyses

The difference between extraction methods was tested with Wilcoxon signed rank test (R v. 3.6.0, 2019). P-values less than or equal to 0.05 were considered significant. qPCR without amplification (NA, not amplified) in at least 2 of 3 replicate reactions was set to a Ct of 40. The qPCR sensitivity for PB samples was compared to the assumed population sensitivity of 50% with two-tailed exact binomial test (R v. 3.6.0, 2019). ggplot2 v. 3.2.1 R package was used for data visualization.

## Results

### Analysis of DNA extraction methods for *M*. *leprae* from mice samples

The effect of DNA extraction procedures on the detection of *M*. *leprae* was assessed for clinical samples from leprosy patients as well as an experimental murine model. The quantity of the total extracted DNA was measured by spectrophotometry, then sensitivity for the *M*. *leprae* bacillus target (16S rRNA) and the mammalian target (18S rRNA) were determined. We used the term efficiency to judge the extraction methods’ performance based initially on spectrometry DNA quantification and then on 16S rRNA qPCR Ct results.

**Fig 2:**
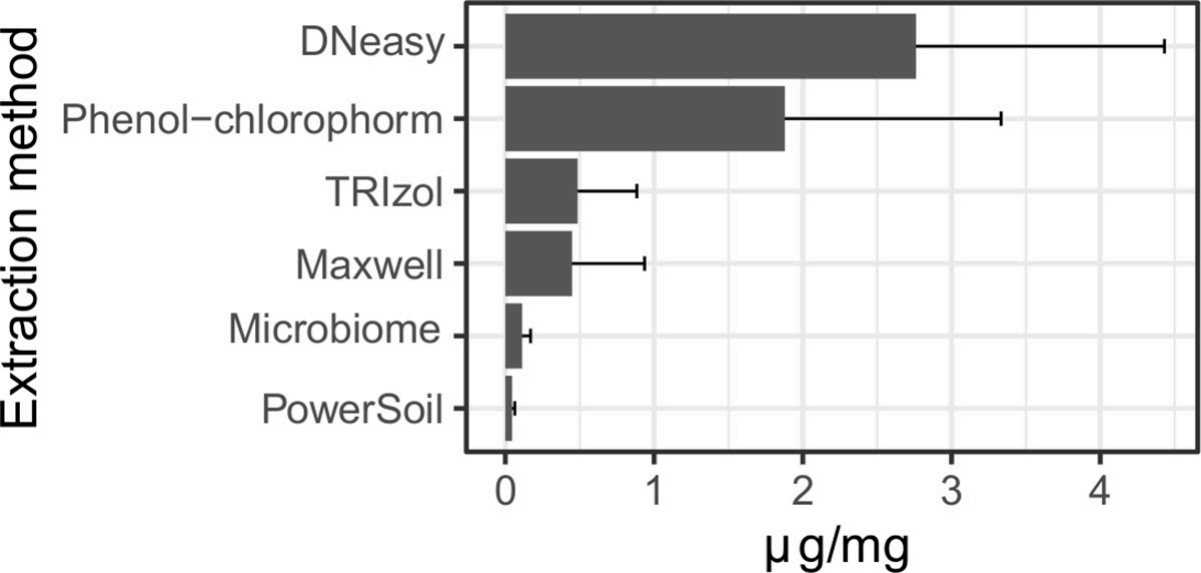
Total DNA yield from a fragment of mouse footpad tissue extracted by six different methods. μg/mg refers to the total extracted DNA per milligram of mouse footpad tissue. Data are displayed as mean plus standard deviation (SD) from mouse batches processed independently. All kits were analyzed in triplicate with the exception of Microbiome, which was analyzed in duplicate.

Four commercial methods (QIAamp DNA Microbiome Kit, DNeasy Blood & Tissue Kit (QIAGEN); Maxwell 16 DNA Purification Kit (Promega); PowerSoil DNA Isolation Kit (MoBio) and two other in-house DNA extraction approaches (phenol-chloroform method and TRIzol/FastPrep system) were tested with three independent sample batches of mouse footpads previously infected with *M*. *leprae*. Of the six tested methods, on average, the DNeasy Blood & Tissue Kit and the phenol-chloroform method recovered the highest quantities of DNA, at 2.76 μg/mg and 1.87 μg/mg, respectively (Fig 2). Whilst the PowerSoil (0.04 μg/mg) and QIAamp DNA Microbiome (0.11 μg/mg) kits recovered the lowest amounts of total DNA (Fig 2, S2 Table). The integrity of the extracted DNA was evaluated spectrophotometrically (A_260_/A_280_ and A_260_/A_230_ ratios) and showed no specific trend regarding initial material amount or final elution volume. Observed absorbance ratios at A_260_/A_280_ were within the expected range for good-quality DNA (1.7–2.0). Although the ratios at A_260_/A_230_ were suboptimal with some values below 1.5, we observed no significant inhibition of the downstream application (detection of host gene by qPCR) (S2 Table).

**Fig 3:**
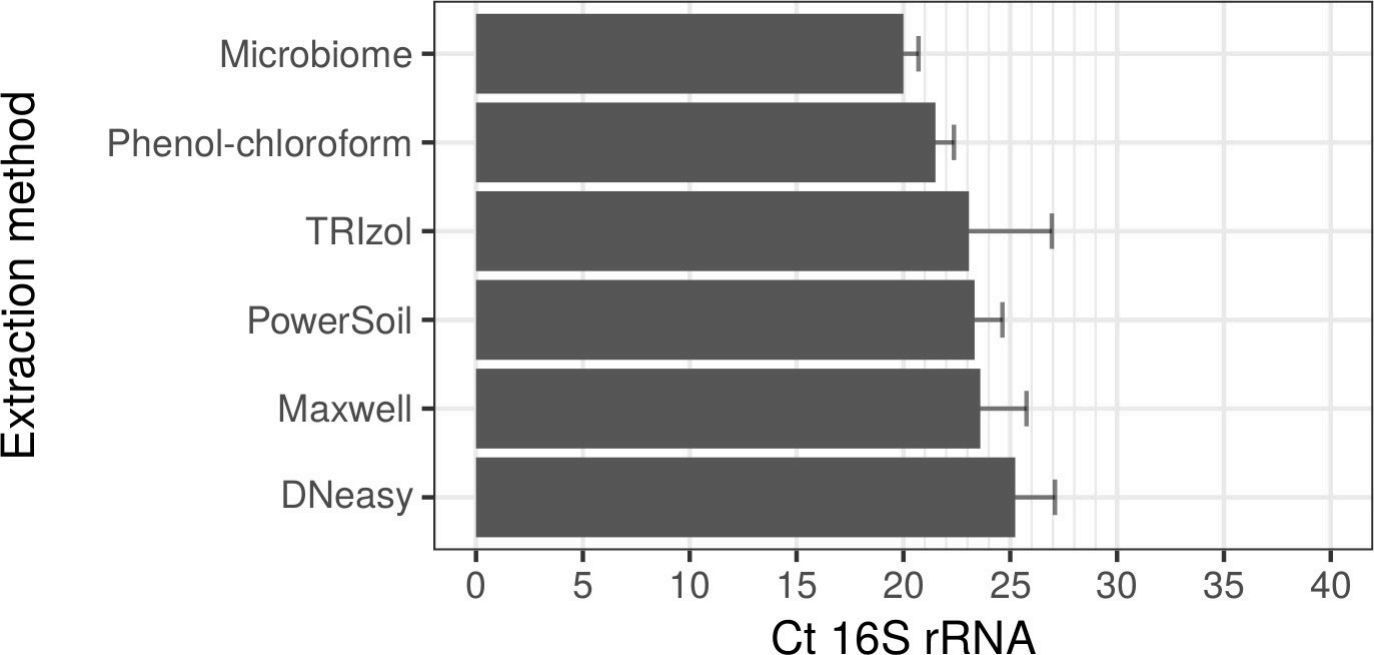
Ct values for *M*. *leprae* target (16S rRNA) amplified by qPCR from infected mouse footpad tissue extracted by different DNA extraction procedures. Data are displayed as mean plus standard deviation (SD) from batches processed independently. All kits were analyzed in triplicate with the exception of Microbiome, which was analyzed in duplicate.

As well as being the most efficient regarding total DNA yield, *M*. *leprae* DNA in the sample extracted by the DNeasy kit was detected well by duplex qPCR (Fig 3). Whilst the sample extracted with the Microbiome kit, which resulted in a very low total DNA yield, performed the best in the qPCR for detection of *M*. *leprae* DNA. This sample had a mean 16S rRNA Ct difference of 5.23 and, consequently detected, on average, 17 times more *M*. *leprae* genomes than in the sample extracted by DNeasy.

Based on these results, the QIAamp DNA Microbiome Kit and the in-house phenol-chloroform approach were the two most efficient methods, with 16S rRNA Ct averages across batches of 20.0 (1.4504 × 10^7^ *M*. *leprae* genomes per μg of tissue DNA) and 21.5 (5.354 × 10^6^ *M*. *leprae* genomes per μg of tissue DNA), respectively (Fig 3, S2 Table). The difference between the Ct mean values for the Microbiome and phenol-chloroform extracted samples is less than 2 Cts or the equivalent to 9.1494 × 10^6^ *M*. *leprae* genomes per μg of tissue DNA. This suggests that, regardless of the lower total DNA extraction yield, the Microbiome kit consistently resulted in a better detection of *M*. *leprae* DNA by qPCR.

**Fig 4:**
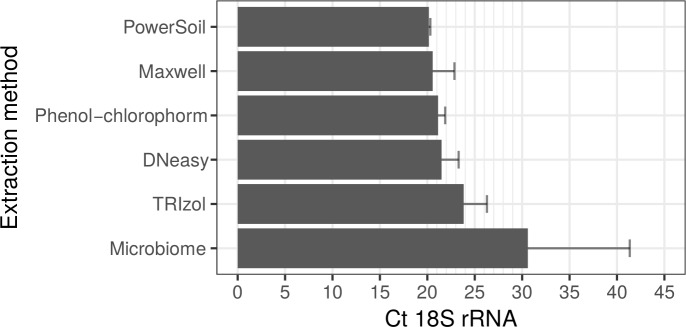
Ct values for mammalian target (18S rRNA) amplified by qPCR from infected mouse footpad tissue extracted by different DNA extraction procedures. Data are displayed as mean plus standard deviation (SD) from batches processed independently. All kits were analyzed in triplicate with the exception of Microbiome, which was analyzed in duplicate.

Samples obtained from most of the DNA extraction kits resulted in similar levels of efficiency regarding amount of total host (mostly mice) DNA detected in duplex qPCR, as shown by similar Ct values for the host 18S rRNA target (Fig 4, S2 Table). The exception to this was the QIAamp DNA Microbiome Kit, which exhibited a loss of internal control amplification compared to other extraction methods, such as the DNeasy Blood & Tissue Kit. This was to be expected as the Microbiome kit involves a host DNA degradation step prior to cell wall lysis of the microorganism leading to enrichment of mycobacterial DNA and reduced quantities of host DNA.

### Analysis of two DNA extraction methods for different biological samples of leprosy patients

We next assessed the efficiency of two of these extraction methods on patient samples. For this, we chose to test the kit that produced samples that were the most efficient for the detection of *M*. *leprae* DNA from infected mice, the QIAamp DNA Microbiome Kit, and compared it to the other commercial method, the DNeasy Blood & Tissue Kit, which produced the greatest amounts of total DNA and has routinely been used with samples from untreated leprosy patients [14,27].

Eight different types of biological clinical samples [whole blood (WB), skin scraping (SS), skin biopsy (SB), skin lesion swab (LS), oral swab (OS), nasal swab (NS), body hair (HR) and blood on FTA paper (FTA)] were collected in 47 patients evaluated with different leprosy clinical forms (23 MB and 24 PB) attended at the Fiocruz leprosy Outpatient Clinic (S1 Table). Not all samples were collected for each of the patients due to technical issues. For example, the total number of body hair samples tested is less than for the other sample types, because some individuals had no body hair to collect around the lesion.

**Fig 5:**
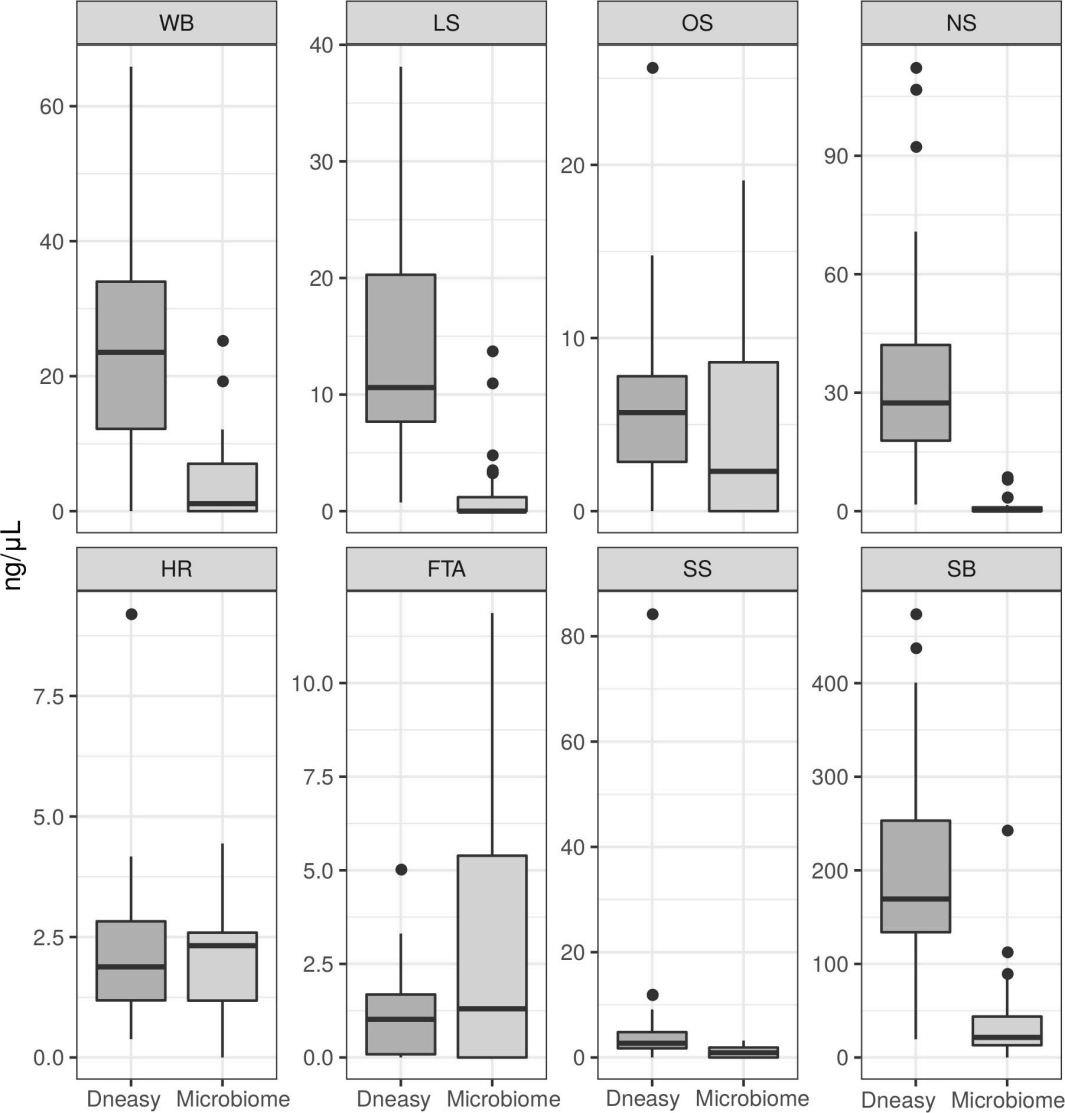
Total DNA yields from patient samples extracted by two different methods. Raw data displayed from spectrophotometer quantification. Boxplots show median, first quartile, third quartile, whiskers (1^st^ or 3^rd^ quartiles ± 1.5 × IQR) and outliers. SB, skin biopsy; SS, skin scraping; NS, nasal swab; HR, body hair; FTA, blood on FTA paper; LS, skin lesion swab; WB, whole blood; OS, oral swab; MB, multibacillary leprosy; PB, paucibacillary leprosy.

As observed with the experiments performed with DNA extracted from infected mouse footpads, for most patient samples the DNeasy kit presented a greater median concentration of total DNA compared to the Microbiome kit. The clinical sample type that resulted in the highest median concentration of extracted DNA was the skin biopsy, for both MB and PB cases, at 169.0 ng/μL (IQR 119.0) and 21.5 ng/μL (IQR 30.7) for DNeasy and Microbiome, respectively (Fig 5, S3 Table).

These extraction methods were then assessed in terms of *M*. *leprae* DNA detection for each of the different clinical samples. Since DNA concentration directly influences its detection, the same concentration (50 ng of total DNA mass) was used across all duplex qPCR experiments.

**Fig 6:**
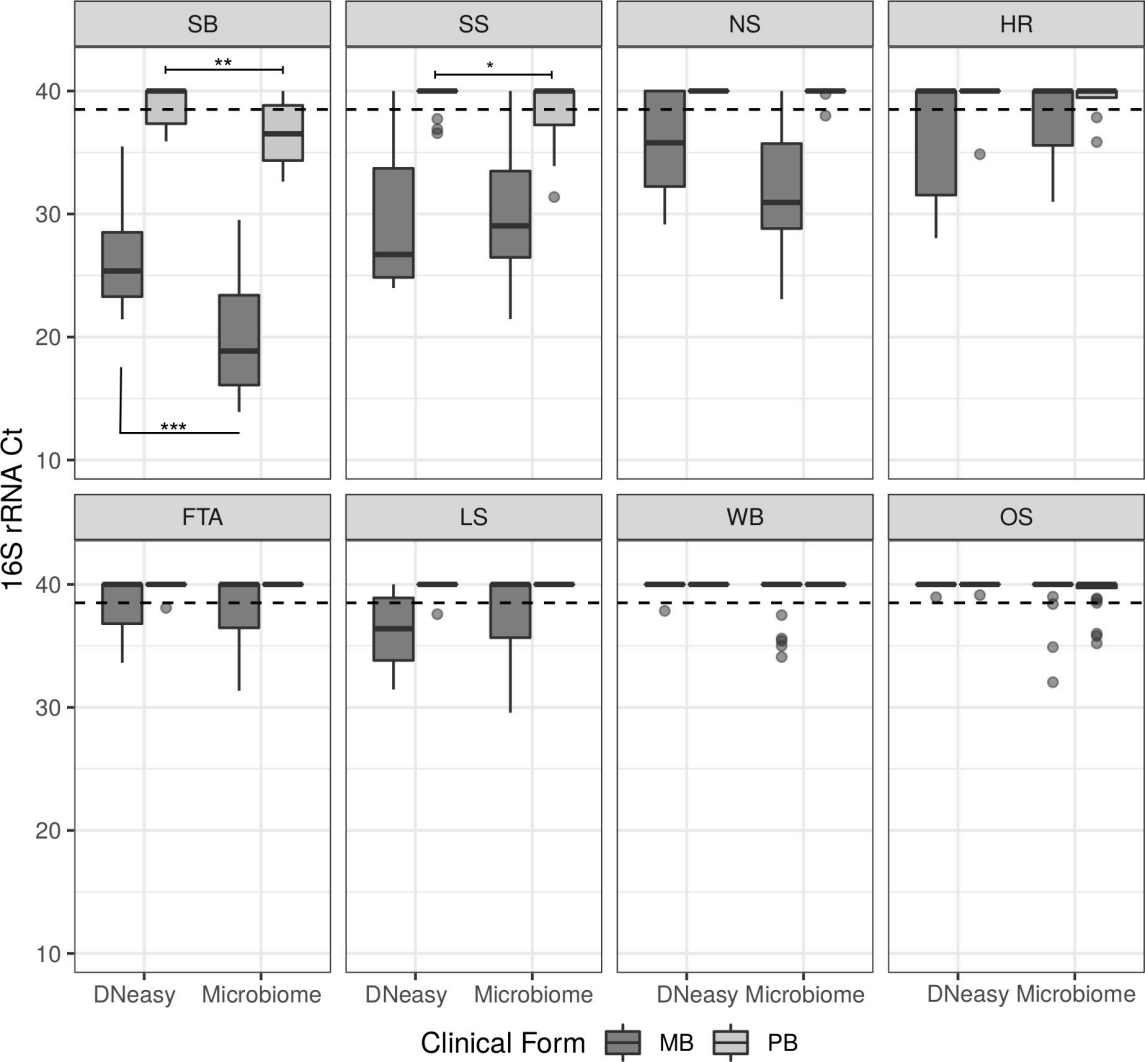
Ct distribution for *M*. *leprae* target (16S rRNA) from different patient sample types extracted by different DNA extraction procedures. Boxplots show median, first quartile, third quartile, whiskers (1^st^ or 3^rd^ quartiles ± 1.5 × IQR) and outliers. SB, skin biopsy; SS, skin scraping; NS, nasal swab; HR, body hair; FTA, blood on FTA paper; LS, skin lesion swab; MB, multibacillary leprosy; PB, paucibacillary leprosy. Dashed line intercepts y axis at 38.5. Asterisks illustrate P-values, where: *** < 0.0001, ** < 0.001 and * <0.05.

In Fig 6 and S2 Fig, it is possible to observe that, in fact, the clinical samples from MB patients have the smallest 16S rRNA Ct values compared to PB, meaning the largest number of bacilli genomes detected. The clinical sample type with largest number of *M*. *leprae* genomes detected was the skin biopsy, with 2.47 × 10^4^ to 8.54 × 10^8^ *M*. *leprae* genomes per μg of tissue DNA in the Microbiome-extracted sample, and 4.40 × 10^2^ to 4.53 × 10^6^ *M*. *leprae* genomes per μg of tissue DNA for the DNeasy (S3 Table). On the other hand, for the PB skin biopsy samples extracted with the Microbiome kit, the smallest *M*. *leprae* genomes per μg of tissue DNA detected was 6 × 10^1^ and the largest was 3.06 × 10^3^; while for the DNeasy, the smallest number of *M*. *leprae* genomes per μg of tissue DNA was 1.2 × 10^2^ and the largest 3.4 × 10^2^. Regardless of the extraction kit and clinical form, most of oral swab and whole blood samples had undetermined Cts during qPCR.

We also tested the paired difference in the Ct distributions for both kits separately for the MB and PB forms. In skin biopsy samples, for both PB and MB, the median Ct values for the Microbiome kit were significantly smaller than those for the DNeasy kit (Fig 6). In PB skin biopsies, the median 16S rRNA Ct difference between Microbiome and DNeasy was -3.05, 95% CI [-4.27, -2.02], P-value < 0.001. Whilst for the MB skin biopsies, the median difference was -6.71, 95% CI [-7.36, -5.98], P-value < 0.0001 (Fig 6).

Interestingly, for skin scrapings, which were the second best clinical sample type for *M*. *leprae* detection, only PB samples had a statistically significant difference in Ct distributions when comparing the two kits, with samples extracted with the Microbiome kit performing better. For PB cases of this sample type extracted with Microbiome kit had a -3.36 median Ct difference when compared to DNeasy, 95% CI [-6.47, -0.20], P-value = 0.029.

In Table 1 we summarize the results regarding the percentage of both *M*. *leprae* and host PCR sensitivity for each type of clinical sample extracted by the two different commercial kits.

**Table 1:** PCR sensitivity across clinical forms, kits and targets. qPCR sensitivity (%) for the different tested targets, *M leprae* 16S rRNA and mammalian 18S rRNA, for each of the different biological samples obtained by the two different DNA extraction methods (DNeasy Blood & Tissue Kit and QIAamp DNA Microbiome Kit). **Legend:** QM: QIAamp DNA Microbiome Kit; B&T: DNeasy Blood & Tissue Kit).

			16S rRNA			18S rRNA				16S rRNA			18S rRNA		
Sample	Clinical Form	kit	qPCR			qPCR			kit	qPCR			qPCR		
			**Pos**	**Neg**	**%Sens**	**Pos**	**Neg**	**%Sens**		**Pos**	**Neg**	**%Sens**	**Pos**	**Neg**	**%Sens**
Skin biopsy	MB	QM	23	0	100%	22	1	96%	B&T	23	0	100%	23	0	100%
Skin scraping	MB	QM	21	2	91%	1	22	4%	B&T	21	2	91%	18	5	78%
Nasal swab	MB	QM	20	3	87%	2	21	8%	B&T	15	8	65%	23	0	100%
Blood FTA	MB	QM	11	12	48%	20	3	87%	B&T	9	14	40%	21	2	91%
Lesion swab	MB	QM	10	13	43%	0	23	0%	B&T	17	6	74%	3	20	13%
Whole Blood	MB	QM	4	19	17%	2	21	9%	B&T	1	22	4%	20	3	87%
Oral swab	MB	QM	3	20	13%	1	22	4%	B&T	0	23	0%	23	0	100%
Body hair	MB	QM	6	7	46%	1	12	8%	B&T	6	7	46%	5	8	38%
Skin biopsy	PB	QM	15	5	75%	15	5	75%	B&T	6	14	30%	20	0	100%
Skin scraping	PB	QM	7	17	29%	2	22	8%	B&T	3	21	13%	20	4	83%
Nasal swab	PB	QM	1	23	4%	4	20	17%	B&T	0	24	0%	24	0	100%
Blood FTA	PB	QM	0	24	0%	21	3	87%	B&T	1	23	4%	23	1	96%
Lesion swab	PB	QM	0	24	0%	0	24	0%	B&T	1	23	4%	8	16	33%
Whole Blood	PB	QM	0	24	0%	5	19	20%	B&T	0	24	0%	24	0	100%
Oral swab	PB	QM	3	21	13%	2	22	8%	B&T	0	24	0%	22	2	92%
Body hair	PB	QM	2	6	25%	2	6	25%	B&T	0	10	0%	4	6	40%

The skin biopsy was the clinical sample that presented the highest sensitivity for *M*. *leprae* DNA by qPCR for the two types of extraction method used. For MB samples using both extraction kits, 100% of samples were positive. Whereas for the PB cases, 75% were positive when extracted with the Microbiome kit and 30% with the DNeasy kit. This indicates that although skin biopsies are the most invasive sample, it is the best for bacillus DNA detection from patients with leprosy (Table 1, Fig 5).

The sensitivity for MB patients varied according to the clinical sample type, where skin scrapings had 91% sensitivity for both kits; nasal swabs 87% sensitivity for the Microbiome kit and 65% for the DNeasy kit; and lesion swabs 43% and 74% for the Microbiome and DNeasy kits, respectively. For PB patients, the Microbiome kit was more efficient in detecting *M*. *leprae* DNA in almost all types of biological samples. Skin biopsy and skin scraping were the clinical samples with the highest sensitivity (75% and 29%, respectively). Interestingly, for skin biopsy samples the Microbiome kit had a 45% increased sensitivity in PB cases than the DNeasy kit (Table 1).

The Microbiome kit resulted in loss of host DNA, with only the skin biopsy and blood on FTA cards showing high sensitivity of the 18S rRNA. In the case of the skin biopsy, this was probably due to an excess of host DNA present that was not totally degraded by the enzymatic step in this kit. While for the FTA samples, the cellulose in the FTA card may have interfered with the enzymatic degradation. As for the DNeasy kit, most sample types successfully amplified the 18S rRNA internal control, with the exception of lesion swab and body hair, where less than 50% were positive for this target (Fig 6, Table 1).

## Discussion

Although there is a need for a standardized and optimized molecular diagnostic assay for leprosy, most studies are focused on creating new detection methodologies or improving those that currently exist, or identifying new targets [28–32], rather than enhancing the sample preparation for use in these methods. The pre-analytical DNA extraction step from the biological sample is considered critical for nucleic acid detection assays, as the yield and purity can affect the sensitivity and specificity in the chosen detection method [33].

Here, we tested the different methods of extraction on the detection of *M*. *leprae* DNA in clinical samples typically collected from suspected leprosy patients. Although molecular detection of *M*. *leprae* from less invasive clinical samples would be ideal, such as skin, oral and nasal swabs, or blood on FTA cards, skin biopsies and skin scrapings still appear to be the best type of specimen samples for pathogen detection, especially from patients with lower bacterial loads, such as in paucibacillary (PB) cases. Nevertheless, using the correct extraction method could mean a reduction in the size of the collected biopsy for *M*. *leprae* detection.

Skin biopsies, skin scrapings, peripheral blood and nasal swab samples have already been evaluated by different groups for leprosy diagnosis [18,34,35]. Araújo and cols. [34] used RLEP qPCR to detect *M*. *leprae* comparing the efficiency using clinical samples from 32 PB and 81 MB patients. The sensitivity for *M*. *leprae* DNA detection of the PB and MB patients respectively was 43.8% and 75.3% for nasal swabs, 9.4% and 74.1% for biopsies, 3.1% and 25.9% for peripheral blood, and 6.2% and 77.8% for skin scrapings [34]. This is similar to what was observed in our study, where we used 16S qPCR, with the skin biopsies, scrapings and nasal swabs presenting good sensitivity for MB, however, we also saw a high sensitivity for the skin biopsies and scrapings of PB patients.

While less invasive samples are desirable and were evaluated in the present work, it is noteworthy that skin biopsies are still the best sample type for *M*. *leprae* 16S rRNA detection using qPCR. Besides being one of the leprosy active sites, possibly with the greatest bacterial load, skin biopsies are the sample type with the highest biological material available for extraction procedures.

Although most studies use RLEP (multi-copy target) due to its greater sensitivity, in this work we chose to use the 16S rRNA single copy target because it presents higher specificity based on the group's previous results [12]. Besides that, Gurung et al. [36] in their systematic review corroborated that PCR assays targeting RLEP have not shown a different sensitivity from those not targeting RLEP.

*M*. *leprae* detection in DNA extracted from lesion swabs using the DNeasy Blood & Tissue Kit was 74% whilst an 87% detection rate was observed in the nasal swab samples extracted using the QIAamp DNA Microbiome Kit. This data suggests the possibility of using these sample types in analysis for suspected relapse and/or drug resistance cases precluding the use of a skin biopsy. Since swabs are compact and provide room temperature stability, they can be easily shipped and are suitable for field sample collection, which could aid in resistance surveillance strategies.

Our assays with peripheral blood showed that this sample was not suitable to detect *M*. *leprae* in both MB and PB cases, as have already verified by others [17,34]. This phenomenon may be explained by the fact that the bacillus is presumably only detected in blood during the short period where it migrates to the extremities, which is the location of optimum conditions for bacteria survival [17]. Interestingly, blood stored on FTA cards and extracted with the Microbiome kit showed a sensitivity of about 50% in MB patients, but the same was not seen in PB patients.

While general bacterial DNA extraction methods have been rigidly investigated in the past, few have focused specifically on *M*. *leprae*. According to many studies targeting diverse microbiota [23], mechanical lysis (repeated bead-beating) is critical for greater bacterial richness in humans samples due to the rigid bacterial cell walls. A combination of enzymatic and mechanical cell lysis (repeated bead-beating in lysis buffer) can also help increasing the purity of extracted bacterial DNA. In this study, we employed an enzymatic-mechanical lysis method to current bacterial DNA extraction kits to investigate the yield and amplification detection of bacterial DNA from distinct biological samples.

Overall, the data presented in this study indicate that the choice of extraction kit can affect the amount of recovered nucleic acids from the pathogen and host, thereby affecting detection methods. The QIAamp DNA Microbiome Kit led to successful detection of *M*. *leprae* DNA, greater than 40%, in almost all types of biological samples collected from MB patients, except for lesion swabs. In PB patients, whose bacilli DNA availability is scarcer, the only biological sample that showed sensitivity for the 16S rRNA target greater than 40% was the skin biopsy using the same extraction method. The PCR sensitivity obtained when using the DNeasy kit were smaller than those previously reported in our routine molecular diagnostic test for PB patients using this kit, 30% against 50% [13,14]. However, due to the small sample size used in this study we cannot conclude that we have found a different population sensitivity (Exact binomial test P-value 0.1153–95% CI [11.89%, 54.28%]). Enrichment of bacterial DNA occurs in the QIAamp DNA Microbiome Kit due to host DNA depletion during the purification process, as compared to the classical DNA extraction methods involving detergents and proteinase K. This crucial step enables more potential in the detection of PB cases, which are harder to clinically diagnose. The diagnostic approaches could benefit with this improved sensitivity. Therefore, we suggest that spectrophotometric total DNA concentration is not a predictor for how an extraction procedure is going to affect qPCR detection of a specific *M*. *leprae*, as long as it is within acceptable good-quality ranges.

Our results show that some isolation procedures might improve qPCR *M*. *leprae* detection, but more studies are needed since several factors affect qPCR testing in a diagnostic setting. Previous case-control and cohort investigations showed that several factors influence diagnostic metrics; e.g., biological sample isolation procedure, molecular target choice, lack of gold-standard tests, sample collection and storage, and analytical reagents. Therefore, all this need to be taken into account before generalizing our results to clinical implementation straightway [36].

The results found with QIAamp DNA Microbiome Kit were very promising, but this kit is still an expensive product. Therefore, in order to implement this extraction as routine in developing world leprosy research centers, understanding the steps of the method that lead to this increased *M*. *leprae* DNA yield and detection would be required to generate a more cost-effective alternative. The results of this study indicate that by increasing the efficiency of the sample extraction and subsequent bacilli detection, thereby reducing test invasiveness, lead us one step closer to a gold standard molecular test for the diagnosis of leprosy.

## Supporting information

S1 Text Protocols of DNA extraction. (PDF)

S1 Fig Standard curve of the amplification of *M*. *leprae* 16S rRNA. DNA template ranged from 1 ng to 5 fg. Ct = Cycle threshold, Rho = correlation coefficient, eff = amplification efficiency.
(TIF)

S2 Fig Individual Ct values for *M*. *leprae* target (16S rRNA) from different types of patient samples extracted by different DNA extraction procedures. NA, not amplified; SB, skin biopsy, SS, skin scraping; NS, nasal swab; HR, body hair; FTA, blood on FTA paper; LS, skin lesion swab; WB, peripheral whole blood; OS, oral swab; MB, multibacillary leprosy; PB, paucibacillary leprosy. Blue line intercepts y axis at 38.5.
(TIF)

S1 Table Sociodemographic and laboratory variables for leprosy patients included in this study. (XLSX)

S2 Table Results of analysis of six DNA extraction methods from *M*. *leprae*-infected footpads of athymic nude mice. (XLS)

S3 Table Results of analysis of two DNA extraction methods for different biological samples of leprosy patients. (XLS)
