# Supplementary material for: Ultra-sensitive detection of Mycobacterium leprae: DNA extraction and PCR assays
Source: PLoS Negl Trop Dis. 2020 May 26;14(5):e0008325. doi: 10.1371/journal.pntd.0008325 (PMC7274454; doi:10.1371/journal.pntd.0008325)
Supplement: S1 Text — (PDF) [file pntd.0008325.s001.pdf]

PROTOCOLS OF DNA EXTRACTION FROM *M. LEPRAE*-INFECTED FOOTPADS OF ATHYMIC *NUDE* MICE

M. leprae DNA isolation from infected mouse footpads was tested with six DNA extraction methods: DNeasy Blood & Tissue Kit (QIAGEN), QIAamp DNA Microbiome Kit (QIAGEN), Maxwell 16 DNA Purification Kit (Promega), PowerSoil DNA Isolation Kit (QIAGEN/Mo Bio), *in-house* standard phenol-chloroform (Sigma Aldrich) plus FastPrep Lysing Matrix B (MP Biomedicals) and TRIzol (Thermo Fisher Scientific).

1. Kit: DNeasy Blood and Tissue (QIAGEN)

Protocol: Purification of Total DNA from Animal Tissues

1. Cut up to 25 mg tissue into small pieces, and place in a 1.5 ml microcentrifuge tube;
2. Add 180 µl Buffer ATL;
3. Add 20 µl proteinase K. Mix thoroughly by vortexing and incubate at 56 °C until the tissue is completely lysed (approximately 3 hours). Vortex occasionally during incubation to disperse the sample, or place in a thermomixer, shaking water bath, or on a rocking platform;
4. Vortex for 15 s. Add 200 µl Buffer AL to the sample and mix thoroughly by vortexing. Then add 200 µl ethanol (96–100%), and mix again thoroughly by vortexing;
5. Pipet the mixture from step 4 (including any precipitate) into the DNeasy Mini spin column placed in a 2 ml collection tube (provided). Centrifuge at 6000 x g (8000 rpm) for 1 min. Discard flow-through and collection tube;
6. Place the DNeasy Mini spin column in a new 2 ml collection tube (provided), add 500 µl Buffer AW1, and centrifuge for 1 min at 6000 x g (8000 rpm). Discard flow-through and collection tube;
7. Place the DNeasy Mini spin column in a new 2 ml collection tube (provided), add 500 µl Buffer AW2, and centrifuge for 3 min at 20,000 x g (14,000 rpm) to dry the DNeasy membrane. Discard flow-through and collection tube;
8. Place the DNeasy Mini spin column in a clean 1.5 ml or 2 ml microcentrifuge tube (not provided), and pipet 100 µl Buffer AE directly onto the DNeasy membrane. Incubate at room temperature for 1 min, and then centrifuge for 1 min at 6000 x g (8000 rpm) to elute;
9. Extracted DNA is stored at 4º C.

2. QIAamp DNA MICROBIOME (QIAGEN)

1. Cut up to 25 mg tissue into small pieces, add 250 µl Buffer AHL in a 2 ml tube (not provided) and incubate for 30 min at room temperature with end-over-end rotation;
2. Centrifuge the tube at 10,000 x g for 10 min and carefully remove the supernatant;
3. Add 190 µl Buffer RDD and 2.5 µl Benzonase. Mix well and incubate at 37 °C for 30 min at 600 rpm in a heating block or water bath;
4. Add 20 µl Proteinase K and incubate at 56°C for 60 min at 600 rpm in a heating block or water bath;
5. Briefly spin the tube at slow speed to remove condensation. Add 200 µl Buffer ATL (containing Reagent DX). Mix well to avoid loss of sample material and transfer into a Pathogen Lysis Tube L;
6. Place the Pathogen Lysis Tube L into a FastPrep-24 instrument. Apply a velocity of 6.5 m/s twice for 45 s each with a 5-min interval during which the samples should be stored on ice;
7. Centrifuge the Pathogen Lysis Tube L at 10,000 x g for 1 min to reduce the amount of foam after lysis. Mix carefully and transfer the supernatant to a fresh microcentrifuge tube;
8. Add 40 µl Proteinase K, mix by vortexing, and incubate at 56 °C for 60 min at 600 rpm in a heating block or water bath;
9. Add 200 µl Buffer APL2. Mix by pulse vortexing for 30 s;
10. Incubate at 70 °C for 10 min and briefly spin the tube;
11. Add 200 µl ethanol to the lysate. Mix thoroughly by pulse vortexing for 15–30 s;
12. Carefully apply up to 700 µl of the mixture from step 11 to the QIAamp UCP Mini Column without wetting the rim. Close the cap and centrifuge at 6,000 x g for 1 min;
13. Discard the flow-through. Put the column back into the collection tube to repeat step 12 with any remaining mixture from step 11;
14. Transfer the QIAamp UCP Mini Column to a fresh collection tube. Carefully open the cap and add 500 µl Buffer AW1 without wetting the rim. Close the cap and centrifuge at 6,000 x g for 1 min. Place the QIAamp UCP Mini Column into a fresh 2 ml collection tube and discard the filtrate;
15. Carefully open the QIAamp UCP Mini Column and add 500 µl Buffer AW2 without wetting the rim. Centrifuge at full speed (20,000 x g) for 3 min;

16. Place the QIAamp UCP Mini Column into a fresh 2 ml collection tube. Discard the filtrate. Centrifuge at full speed (20,000 x g) for 1 min;
17. Place the QIAamp UCP Mini Column into a fresh 1.5 ml tube and apply 50 µl Buffer AVE directly onto the center of the membrane. Close the lid and incubate at room temperature for 5 min;
18. Centrifuge at 6,000 x g for 3 min to elute the DNA;
19. Extracted DNA is stored at 4º C.

3. Maxwell 16 TISSUE DNA Purification Kits (Promega)

1. The Maxwell 16 Instrument must be configured with the Maxwell 16 SEV Hardware Kit;
2. Place each cartridge to be used into the holder with the ridged side of the cartridge facing toward the numbered side of the rack. Remove the seal from each cartridge;
3. Place one plunger into well #7 of each cartridge such that the bottom of the plunger is at the bottom of the cartridge (Well #7 is the well closest to the ridged side of the cartridge.);
4. Place tissue (up to 50mg) into well #1 of predispensed cartridge (Well #1 is the well closest to the cartridge label and furthest from the user.);
5. Verify that the instrument mode is set to Research. Verify that“Rsch” and “SEV” are displayed as shown;
6. Use the Scroll Up or Scroll Down button to move the cursor to “Run” to perform a purification run. Press “Run/Stop” to select;
7. Use the Scroll Up or Scroll Down button to move the cursor to “DNA”. Press “Run/Stop” to select;
8. Use the Scroll Up or Scroll Down button to move the cursor to the purification method/sample type. Sample Type: DNA and Protocol: tissue. Use the Scroll Up or Scroll Down button to move the cursor to “OK”. Press the “Run/Stop” button to continue with a purification run;
9. Open the door when prompted to do so on the LCD display. Press the “Run/Stop” button to extend the platform out of the instrument for easy insertion of the cartridges;
10. Transfer cartridges containing samples and plungers from the cartridge preparation rack onto the Maxwell 16 platform;
11. Place one blue Elution Tube for each cartridge into the Elution Tube slots at the front of the platform;

12. Add 300µl of Elution Buffer to each blue Elution Tube;
13. Press the “Run/Stop” button. The platform will retract. Close the door;
14. The Maxwell 16 Instrument will begin the purification run. The LCD screen displays the steps performed and approximate time remaining in the run;
15. When purification is complete, the LCD screen will display a message that the method has ended. Upon completion, open the instrument door. Check to make sure that all plungers have been removed from the magnetic rod assembly. If the plungers have not been removed, push them down gently by hand to remove them;
16. Press the “Run/Stop” button to extend the platform out from inside the instrument;
17. Remove the Elution Tubes from the platform-heated Elution Tube slots, and place them into the Magnetic Elution Tube Rack. Allow the residual magnetic particles to collect on the magnetized side of the tube. The amount of particles will vary with sample size and composition;
18. Transfer the eluted samples into the storage tube by pipetting. Note: To avoid particle transfer, use a pipette tip to aspirate samples away from the captured particles on the side of the blue Elution Tube;
19. Remove cartridges and plungers from the instrument platform, and discard them.

4. PowerSoil DNA Isolation Kit (MO BIO)

1. Cut up to 25 mg tissue into small pieces, and place in PowerBead tube;
2. Gently vortex to mix;
3. Add 60µl of Solution C1 and invert several times or vortex briefly;
4. Secure PowerBead Tubes horizontally using the MO BIO Vortex Adapter tube holder for the vortex (MO BIO Catalog No. 13000-V1) or secure tubes horizontally on a flat-bed vortex pad with tape. Vortex at maximum speed for 10 minutes;
5. Make sure the PowerBead Tubes rotate freely in your centrifuge without rubbing. Centrifuge tubes at 10,000 x g for 30 seconds at room temperature. CAUTION: Be sure not to exceed 10,000 x g or tubes may break;
6. Transfer the supernatant to a clean 2 ml Collection Tube (provided). Note: Expect between 400 to 500µl of supernatant. Supernatant may still contain some soil particles;
7. Add 250µl of Solution C2 and vortex for 5 seconds. Incubate at 4°C for 5 minutes;

8. Centrifuge the tubes at room temperature for 1 minute at 10,000 x g;
9. Avoiding the pellet, transfer up to, but no more than, 600 µl of supernatant to a clean 2 ml Collection Tube (provided);
10. Add 200µl of Solution C3 and vortex briefly. Incubate at 4 °C for 5 minutes;
11. Centrifuge the tubes at room temperature for 1 minute at 10,000 x g;
12. Avoiding the pellet, transfer up to, but no more than, 750 µl of supernatant into a clean 2 ml Collection Tube (provided);
13. Add 1200 µL of Solution C4 to the supernatant and vortex for 5 seconds;
14. Load approximately 675 µl onto a Spin Filter and centrifuge at 10,000 x g for 1 minute at room temperature. Discard the flow through and add an additional 675µl of supernatant to the Spin Filter and centrifuge at 10,000 x g for 1 minute at room temperature. Load the remaining supernatant onto the Spin Filter and centrifuge at 10,000 x g for 1 minute at room temperature. Note: A total of three loads for each sample processed are required;
15. Add 500µl of Solution C5 and centrifuge at room temperature for 30 seconds at 10,000 x g. Discard the flow through;
16. Centrifuge again at room temperature for 1 minute at 10,000 x g;
17. Carefully place Spin Filter in a clean 2 ml Collection Tube (provided). Avoid splashing any Solution C5 onto the Spin Filter;
18. Add 100 µL of Solution C6 to the center of the white filter membrane. Alternatively, sterile DNA-Free PCR Grade Water may be used for elution from the silica Spin Filter membrane at this step (MO BIO Catalog No. 17000-10);
19. Centrifuge at room temperature for 30 seconds at 10,000 x g;
20. Discard the Spin Filter. The DNA in the tube is now ready for any downstream application. No further steps are required. We recommend storing DNA frozen (-20° to -80°C). Solution C6 contains no EDTA. To concentrate the DNA see the Additional Information Section.

5. Phenol-Cloroform (sigma)/ Fast Prep

1. Cut up to 25 mg tissue into small pieces, and place in a 1.5 ml microcentrifuge tube;
2. Add 400 µL TE [Tris-EDTA: 200mM Tris-HCl, pH 8.0; ethylene diamino tetraacetic acid (EDTA), 5mM, pH 8.0 to the cell precipitate and homogenize by pipetting / vortexing;
3. Transfer contents to a new tube with beads (Lysing matrix B /MP biomedicals) and add 400 µl of saturated phenol;

4. Place the Lysing matrix B into a FastPrep-24 instrument. Apply a velocity of 6.5 m/s for 45 s each with a 5-min interval during which the samples should be stored on ice;

5. Centrifuge for 10 minutes at 14,000 rpm and rapidly transfer the aqueous phase to a new eppendorf tube (Approximately 250 µL);

6. To increase the amount of total DNA extracted, 200 µl of TE was added to the phenol-containing tube (phase 3). Place these tube into a FastPrep for 60s and after centrifuged 10 min at maximum rotation;

7. Transfer the aqueous phase (approximately 200 µL) to a eppendorf tube that already contains 250 µL of the supernatant obtained in the first step of phase 3, totaling about 450 µL;

8. Add the same volume of phenol-chloroform-isoamyl alcohol (25/24/1), mix by inversion and centrifuge at maximum rotation for 10 minutes;

9. Repeat the previous step using chloroform-isoamyl alcohol (24/1);

10. Transfer the aqueous phase to a new eppendorf tube, add 1/10 volumes of 3M sodium acetate and two volumes of 100% ethanol, and incubate for 1 hour at -70 ° C;

11. After incubation centrifuge for 20 minutes at maximum rotation;

12. Discard the supernatant and resuspend the precipitate in 20 µL of TE, followed by overnight incubation at 4 ° C.

6. Trizol (Thermo-fisher)

1. Add 1 ml of TRIzol Reagent per 50–100 mg of tissue to the sample and homogenize using a homogenizer;

2. Incubate for 5 minutes to permit complete dissociation of the nucleoproteins complex;

3. Add 0.2 ml of chloroform per 1 ml of TRIzol Reagent used for lysis, then securely cap the tube;

4. Incubate for 2–3 minutes;

5. Centrifuge the sample for 15 minutes at 12,000 × g at 4°C. The mixture separates into a lower red phenol-chloroform, and interphase, and a colorless upper aqueous phase;

6. Transfer the aqueous phase containing the RNA to a new tube;

7. Transfer the aqueous phase containing the RNA to a new tube by angling the tube at 45° and pippeting the solution out;

8. Add 0.5 ml of isopropanol to the aqueous phase, per 1 ml of TRIzol Reagent used for lysis;
9. Incubate for 10 minutes;
10. Centrifuge for 10 minutes at 12,000 × g at 4°C. Total RNA precipitate forms a white gel-like pellet at the boJom of the tube;
11. Discard the supernatant with a micropipettor;
12. Resuspend the pellet in 1 ml of 75% ethanol per 1 ml of TRIzol Reagent used for lysis. Note: The RNA can be stored in 75% ethanol for at least 1 year at –20°C, or at least 1 week at 4°C.
13. Vortex the sample brieflly then centrifuge for 5 minutes at 7500 × g at 4°C.
14. Discard the supernatant with a micropipettor.
15. Vacuum or air dry the RNA pellet for 5–10 minutes
16. Solubilize the RNA
17. Resuspend the pellet in 20–50 µL of RNase-free water.
18. Incubate in a water bath or heat block set at 55–60°C for 10–15 minutes. Proceed to downstream applications, or store the RNA at –70°C

Isolate DNA

1. Precipitate the DNA;
2. Remove any remaining aqueous phase overlying the interphase. This is critical for the quality of the isolated DNA;
3. Add 0.3 ml of 100% ethanol per 1 ml of TRIzol Reagent used for lysis;
4. Cap the tube, mix by inverting the tube several times;
5. Incubate for 2–3 minutes;
6. Centrifuge for 5 minutes at 2000 × g at 4°C to pellet the DNA;
7. Transfer the phenol-ethanol supernatant to a new tube. The supernatant is used for protein isolation. If needed, and can be stored at –70°C for several months;
8. Resuspend the pellet in 1 ml of 0.1 M sodium citrate in 10% ethanol, pH 8.5, per 1 ml of TRIzol Reagent used for lysis;
9. Incubate for 30 minutes, mixing occasionally by gentle inversion. Note: The DNA can be stored in sodium citrate/ethanol for at least 2 hours;
10. Centrifuge for 5 minutes at 2000 × g at 4°C;
11. Discard the supernatant with a micropipettor;

12. Repeat step 2a–step 2d once. Note: Repeat step 2a–step 2d twice for large DNA pellets (>200 µg);

13. Resuspend the pellet in 1.5–2 ml of 75% ethanol per 1 ml of TRIzol Reagent used for lysis. g. Incubate for 10–20 minutes, mixing occasionally by gentle inversion. Note: The DNA can be stored in 75% ethanol at several months at 4°C;

14. Centrifuge for 5 minutes at 2000 × g at 4°C;

15. Discard the supernatant with a micropipettor;

16. Vacuum or air dry the DNA pellet for 5–10 minutes;

17. Resuspend the pellet in 0.3–0.6 ml of TE (10 mM Tris, 0.1 mM EDTA).

PROTOCOLS OF CLINICAL SAMPLES DNA EXTRACTION

Leprosy patient samples were tested with two chosen kits, DNeasy Blood & Tissue and QIAamp DNA Microbiome. Each sample type from the patient was divided into equal parts to be used in both DNA extraction kits.

The isolation procedures with QIAamp DNA Microbiome were performed according to protocol used on the experimental mouse model with minor modifications described in table below.

The protocols used for DNA extraction for each of the eight clinical sample types using DNeasy Blood & Tissue are described below.

Clinical Samples:

a) Whole Blood;
b) Lesion Swab;
c) Oral Swab;
d) Nasal Swab;
e) Hair;
f) Blood collected in FTA;
g) Skin Scrapping;
i) Skin biopsy.

All swab samples were stored in a 1:1 ratio of Amies Liquid Medium (eSwab solution -COPAN) and 2X stabilization buffer solution (SBS).

Stabilization buffer solution (SBS)		
**Reagent**	**Volume (ml)/mass (g)**	**Final concentration**
**NaCl**	1,461 g	100 mM
**1M Tris HCl pH 7.5**	2,5 ml	10 mM
**0,5M EDTA**	5 ml	10 mM
**10% SDS**	12,5 ml	0,5%
**Proteinase K (10mg/ml)**	2,5 ml	0,1mg/ml
**ddH2O**	227,5 ml	
**Total Volume**	250 ml	

1. **Kit: DNEasy Blood and Tissue (QIAGEN)**

Protocol: Purification of Total DNA from whole blood

1. Add 200 uL of whole blood into a 1.5 ml microcentrifuge tube, and immediately add 100 µl Buffer AL and 10 uL proteinase K;

2. Mix thoroughly by vortexing, and incubate at 56°C for 1 hour. Briefly spin the tube;

3. Add 50 µl of ethanol (96–100%), and mix again thoroughly by vortexing;

4. Incubate at room temperature for 3 minutes. Briefly spin the tube;

5. Pipet the lysate (including any precipitate) into the DNeasy Mini spin column placed in a 2 ml collection tube (provided). Centrifuge at 6000 x g (8000 rpm) for 1 min. Discard flow-through and collection tube;

6. Place the DNeasy Mini spin column in a new 2 ml collection tube (provided), add 500 µl Buffer AW1, and centrifuge for 1 min at 6000 x g (8000 rpm). Discard flow-through and collection tube;

7. Place the DNeasy Mini spin column in a new 2 ml collection tube (provided), add 700 µl Buffer AW2, and centrifuge for 1 min at 6000 x g (8000 rpm). Discard flow-through and collection tube;

8. Place the DNeasy Mini spin column in a new 2 ml collection tube (provided), add 700 µl ethanol (96–100%), and centrifuge for 1 min at 6000 x g (8000 rpm);

9. Centrifuge again at 8000 x g (14000 rpm) for 3 minutes to dry the DNeasy membrane;

10. Place the DNeasy Mini spin column in a clean 1.5 ml or 2 ml microcentrifuge tube (not provided), and incubate at room temperature for 10 min or at 56 °C for 3 min;

11. Pipet 100 µl Buffer TAE/AE directly onto the DNeasy membrane. Incubate at room temperature for 1 min, and then centrifuge for 1 min at 8000 x g (14000 rpm) to elute.

Protocol: Purification of Total DNA from lesion, oral and nasal swabs

1. Cut half of the swab collection surfasse, plus 500 µl of stock solution (ALM + 2X SBS, same sol. as sample was stored) and place it into a new 1.5 ml microcentrifuge tube;

2. Add 500 uL of ATL buffer, 20 µl of proteinase K and incubate at 56 °C for 1 hour, mixing every 15 minutes. Quickly spin the tube;

3. Add 500 µl Buffer AL to the sample, and mix thoroughly by vortexing;

4. Incubate at 70 °C for 10 minutes (mix every 3 min). Quickly spin the tube;

5. Add 250 µl ethanol (96–100%), and mix again thoroughly by vortexing. Spin the tube;

6. Pipet the mixture into the DNeasy Mini spin column placed in a 2 ml collection tube (provided). Centrifuge at 6000 x g (8000 rpm) for 1 min. Discard flow-through and collection tube;

7. Place the DNeasy Mini spin column in a new 2 ml collection tube (provided), add 500 µl Buffer AW1, and centrifuge for 1 min at 6000 x g (8000 rpm). Discard flow-through and collection tube;

8. Place the DNeasy Mini spin column in a new 2 ml collection tube (provided), add 700 µl Buffer AW2, and centrifuge for 3 min at 6000 x g (8000 rpm) to dry the DNeasy membrane. Discard flow-through and collection tube;

9. Add 700 µl ethanol (96–100%), and centrifuge for 3 min at 6000 x g (8000 rpm) to dry the DNeasy membrane;

10. Repeat the centrifugation now at 8000 x g (14000 rpm) for 3 min;

11. Place the DNeasy Mini spin column in a clean 1.5 ml or 2 ml microcentrifuge tube (not provided), and incubate at room temperature for 10 min or at 56 °C for 3 min;

12. Pipet 100 µl Buffer AE directly onto the DNeasy membrane. Incubate at room temperature for 1 min, and then centrifuge for 1 min at 8000 x g (14000 rpm) to elute.

Protocol: Purification of Total DNA from body hair

1. Centrifuge the tube containing the sample stored in ethanol 70% and discard the supernatant;
2. Elute sample/precipitate with 500 µL PBS (1x) for 30 min at 56 °C;
3. Add 3-5 hairs (preferably with the follicle) and 500 µL PBS 1X solution into a 1.5 ml microcentrifuge tube;
4. Add 300 µl of Buffer ATL and 20 uL of proteinase K. Vortex for 10 seconds and incubate at 56 °C for 1 hour (mixing every 15 minutes). Quick spin;
5. Add 300 uL of AL buffer and mix by vortex. Incubate at 70 °C for 10 minutes (mixing every 3 min). Quick spin;
6. Add 150 uL of ethanol (96–100%), and mix again thoroughly by vortexing followed by a quick spin;
7. Pipet the supernatant (including any precipitate) into the DNeasy Mini spin column placed in a 2 ml collection tube (provided). Centrifuge at 6000 x g (8000 rpm) for 2 min. Discard flow-through and collection tube.
8. Place the DNeasy Mini spin column in a new 2 ml collection tube (provided), add 500 µl Buffer AW1, and centrifuge for 1 min at 6000 x g (8000 rpm). Discard flow-through and collection tube.
9. Place the DNeasy Mini spin column in a new 2 ml collection tube (provided), add 700 µl Buffer AW2, and centrifuge for 3 min at 20,000 x g (14,000 rpm) to dry the DNeasy membrane. Discard flow-through and collection tube.
10. Place the DNeasy Mini spin column in a new 2 ml collection tube (provided), add 700 µl of ethanol (96–100%), and centrifuge for 1 min at 20,000 x g (14,000 rpm) to dry the DNeasy membrane. Discard flow-through and collection tube;
11. Place the DNeasy Mini spin column in a clean 1.5 ml or 2 ml microcentrifuge tube (not provided), and incubate at room temperature for 10 min or at 56 °C for 3 min;
12. Pipet 20-50 uL of ATE/AE buffer directly onto the DNeasy membrane. Incubate at room temperature for 1 min, and then centrifuge for 1 min at 6000 x g (8000 rpm) to elute.
13. Repeat the centrifugation for 1 min at 6000 x g (8000 rpm).

Protocol: Purification of Total DNA from FTA

1. Elute 1 FTA disc in 200 uL PBS (1x) overnight at room temperature;

2. In a 1.5 ml microcentrifuge tube, combine 1 FTA disc (10 mm diameter), 200 µL PBS 1X solution (the same from step 1, in which the card have been eluting in) with 280 uL of ATL buffer and 20 uL Proteinase K. Homogenate in vortex for 15 seconds;

3. Incubate at 56 °C for 1 hour agitating from 15 to 15 minutes;

4. Add 300 µl of Buffer ATL and vortex briefly. Incubate at 70 °C for 10 minutes, vortexing for 10 seconds every 3 minutes;

5. Briefly spin and add 150 uL of absolute ethanol. Then, mix thoroughly by vortexing;

6. Add 150 µl ethanol (96–100%), and mix again thoroughly by vortexing followed by a quick spin;

7. Pipet the supernatant into a DNeasy Mini spin column placed in a 2 ml collection tube (provided). Centrifuge at 6000 x g (8000 rpm) for 2 min. Discard flow-through and collection tube;

8. Place the DNeasy Mini spin column in a new 2 ml collection tube (provided), add 500 µl Buffer AW1, and centrifuge for 1 min at 6000 x g (8000 rpm). Discard flow-through and collection tube;

9. Place the DNeasy Mini spin column in a new 2 ml collection tube (provided), add 700 µl Buffer AW2, and centrifuge for 3 min at 20,000 x g (14,000 rpm) to dry the DNeasy membrane. Discard flow-through and collection tube;

10. Place the DNeasy Mini spin column in a new 2 ml collection tube (provided), add 700 µl of ethanol (96–100%), and centrifuge for 1 min at 6000 x g (8000 rpm). Discard flow-through and collection tube;

11. Place the DNeasy Mini spin column in a clean 1.5 ml or 2 ml microcentrifuge tube (not provided), and incubate at room temperature for 10 minutes or at 56 °C for 3 minutes;

12. Add 100 µl Buffer AE directly onto the DNeasy membrane. Incubate at room temperature for 1 min, and then centrifuge for 1 min at 14000 rpm to elute;

13. Repeat centrifugation at 14000 rpm for 1 minute.

Protocol: Purification of Total DNA from Skin Scraping (SS)

1. The first step was to centrifuge 2000 rpm for 10 minutes the tube containing the sample stored in ethanol 70%. Discard the supernatant and allow to dry for 20-30 min. Elute precipitate in 500 uL of PBS (1x) overnight;

2. Use 200 µL of the previously PBS solution. Add 20 µl proteinase K into the tube containing the sample;

3. Add 200 µl Buffer AL. Mix thoroughly by vortexing and incubate at 56 ºC for 1 hour;

4. Then add 200 µl ethanol (96–100%), and mix again thoroughly by vortexing.

5. Pipet the mixture into the DNeasy Mini spin column placed in a 2 ml collection tube (provided). Centrifuge at 6000 x g (8000 rpm) for 1 min. Discard flow-through and collection tube;

6. Place the DNeasy Mini spin column in a new 2 ml collection tube (provided), add 500 µl Buffer AW1, and centrifuge for 1 min at 6000 x g (8000 rpm). Discard flow-through and collection tube;

7. Place the DNeasy Mini spin column in a new 2 ml collection tube (provided), add 500 µl Buffer AW2, and centrifuge for 3 min at 20,000 x g (14,000 rpm) to dry the DNeasy membrane. Discard flow-through and collection tube;

8. Place the DNeasy Mini spin column in a clean 1.5 ml or 2 ml microcentrifuge tube (not provided), and pipet 100 µl Buffer AE directly onto the DNeasy membrane. Incubate at room temperature for 1 min, and then centrifuge for 1 min at 6000 x g (8000 rpm) to elute;

9. Extracted DNA is stored at 4º C.

Protocol: Purification of Total DNA from skin biopsy

1. The first step was to centrifuge 2000 rpm for 10 minutes the tube containing the sample stored in ethanol 70%. Discard the supernatant and allow to dry for 20-30 min. Elute precipitate in 700 uL of PBS (1x) overnight;

2. Place the skin biopsy fragment into a 1.5 ml microcentrifuge tube, and add 180 µl Buffer ATL and 20 uL proteinase K;

3. Mix thoroughly by vortexing, and incubate at 56 °C for 4-6 hours for small fragments or 12 h for larger pieces. Vortex occasionally during incubation to disperse the sample, or place in a thermomixer, shaking water bath, or on a rocking platform;

4. Add 200 µl Buffer AL to the sample, and mix thoroughly by vortexing. Then add 200 µl ethanol (96–100%), and mix again thoroughly by vortexing;

5. Incubate at room temperature for 5 minutes. Briefly spin the tube;

6. Pipet the lysate (including any precipitate) into the DNeasy Mini spin column placed in a 2 ml collection tube (provided). Centrifuge at 6000 x g (8000 rpm) for 1 min. Discard flow-through and collection tube;

7. Place the DNeasy Mini spin column in a new 2 ml collection tube (provided), add 500 µl Buffer AW1, and centrifuge for 1 min at 6000 x g (8000 rpm). Discard flow-through and collection tube;

8. Place the DNeasy Mini spin column in a new 2 ml collection tube (provided), add 700 µl Buffer AW2, and centrifuge for 1 min at 6000 x g (8000 rpm). Discard flow-through and collection tube;

9. Place the DNeasy Mini spin column in a new 2 ml collection tube (provided), add 700 µl ethanol (96–100%), and centrifuge for 1 min at 6000 x g (8000 rpm);

10. Centrifuge again at 8000 x g (14000 rpm) for 3 minutes to dry the DNeasy membrane;

11. Place the DNeasy Mini spin column in a clean 1.5 ml or 2 ml microcentrifuge tube (not provided), and incubate at room temperature for 10 min or at 56 °C for 3 min;

12. Pipet 100 µl Buffer AE directly onto the DNeasy membrane. Incubate at room temperature for 1 min, and then centrifuge for 1 min at 8000 x g (14000 rpm) to elute.

2. **QIAamp DNA MICROBIOME (QIAGEN)**

The first step of this protocol (amount of starting material) varies according to the type of clinical sample as described in the table below.

After item 2, all steps are the same as described above for DNA extraction from *M. leprae*-infected footpads of athymic *nude* mice.

Type of clinical sample	Collection / Storage	Amount of material employed in the extraction process
**Whole Blood**	-	1ml
**Lesion Swab**	Stored in 2 ml of solution*	350 µl
**Oral Swab**	Stored in 2 ml of solution*	350 µl
**Nasal Swab**	Stored in 2 ml of solution*	350 µl
**Hair**	Elute in 500 ml PBS (1x)** for 30 min at 56 °C .	500uL + hair
**FTA**	Elute 1 disc in 500 µl PBS(1x)** overnight	500 µl + disc to proteinase K digestion step
**Skin Scrapping**	Stored in ethanol 70%. Elute precipitate in 500 µl of PBS (1x)	500 µl
**Skin biopsy**	Stored in 70% etanol. Elute precipitate in 700 µl PBS (1x)	½ of biopsy

*COPAN solution + 2x SBS
**Gibco PBS buffers
