# Supplementary figures and images for: Ultra-sensitive detection of Mycobacterium leprae: DNA extraction and PCR assays
Source: PLoS Negl Trop Dis. 2020 May 26;14(5):e0008325. doi: 10.1371/journal.pntd.0008325 (PMC7274454; doi:10.1371/journal.pntd.0008325)

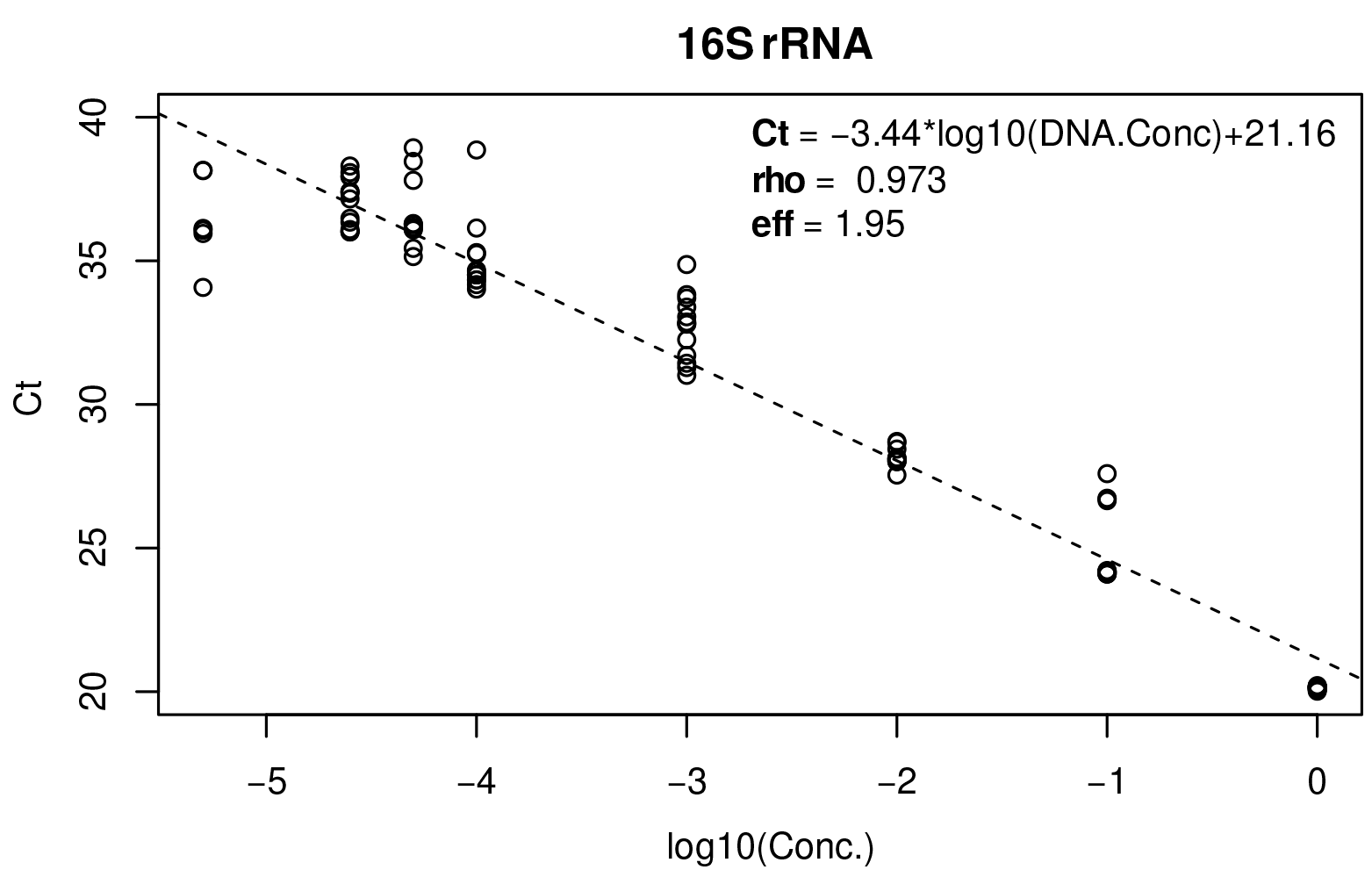

Supplement: S1 Fig — DNA template ranged from 1 ng to 5 fg. Ct = Cycle threshold, Rho = correlation coefficient, eff = amplification efficiency. (TIF) [file pntd.0008325.s002.tif]

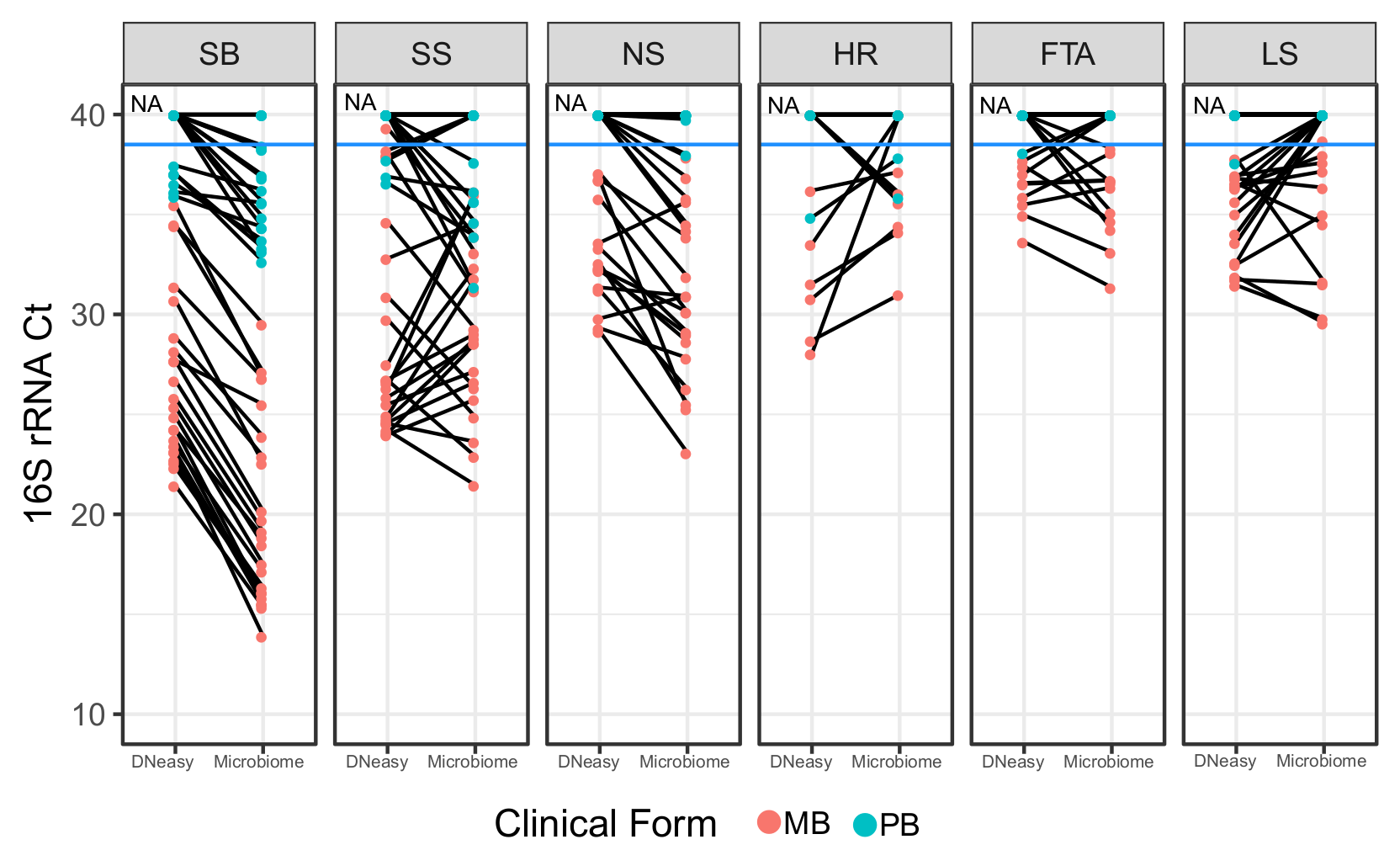

Supplement: S2 Fig — NA, not amplified; SB, skin biopsy, SS, skin scraping; NS, nasal swab; HR, body hair; FTA, blood on FTA paper; LS, skin lesion swab; WB, peripheral whole blood; OS, oral swab; MB, multibacillary leprosy; PB, paucibacillary leprosy. Blue line intercepts y axis at 38.5. (TIF) [file pntd.0008325.s003.tif]
